# Complete Genome Sequence of *Eubacterium hallii* Strain L2-7

**DOI:** 10.1128/genomeA.01167-17

**Published:** 2017-10-26

**Authors:** Sudarshan A. Shetty, Jarmo Ritari, Lars Paulin, Hauke Smidt, Willem M. De Vos

**Affiliations:** a Laboratory of Microbiology, Wageningen University, Wageningen, The Netherlands; b Department of Veterinary Biosciences, Division of Microbiology and Epidemiology, University of Helsinki, Helsinki, Finland; c DNA Sequencing and Genomics Laboratory, Institute of Biotechnology, University of Helsinki, Helsinki, Finland; d RPU Immunobiology, Department of Bacteriology and Immunology, Faculty of Medicine, University of Helsinki, Helsinki, Finland

## Abstract

The complete genome sequence of *Eubacterium hallii* strain L2-7 is reported here. This intestinal strain produces butyrate from glucose as well as lactate when acetate is provided in the growth medium. In addition, strain L2-7 has been shown to improve insulin sensitivity in db/db mice, indicating its application potential.

## GENOME ANNOUNCEMENT

Anaerobic bacteria phylogenetically affiliated with the *Lachnospiraceae* (also known as *Clostridium* cluster XIVa), including species related to *Eubacterium hallii*, make up the majority of highly prevalent bacteria in the human intestinal tract (1, 2). Bacteria related to *Eubacterium hallii* are abundant in the human intestine, belong to the core microbiota, and can produce butyrate from glucose as well as lactate in the presence of acetate (1, 2). To improve our understanding of the genetic potential underlying its metabolic capabilities, the genome of *Eubacterium hallii* strain L2-7, a strain isolated from infant feces and found to improve insulin sensitivity in db/db mice, was sequenced (3, 4).

Genomic DNA of *E. hallii* strain L2-7 (DSM 17630) was extracted using the MasterPure Gram-positive DNA purification kit (Epicentre). The quality of the extracted DNA was measured using a NanoDrop 2000 spectrophotometer (Thermo Scientific, USA). The genome was sequenced using a PacBio RSII instrument, and raw reads were assembled using the PacBio SMRT Analysis pipeline version 2.2 and the HGAP protocol (5). The default settings for genome assembly were used, except for the following adaptations: minimum subread length, 500; minimum polymerase read length quality, 0.80; minimum seed read length, 30; split target into chunks, 1; alignment candidate per chunk, 24; genome size, 3,000,000; target coverage, 30; overlapper error rate, 0.06; overlapper mini length, 40; and overlapper k-mer, 14. The assembled genome was annotated using the SAPP framework (6). This framework consists of variety of tools, including Prodigal version 2.5 (7), InterProScan 5RC7 (8), tRNAscan-SE version 1.3.1, and RNAmmer version 1.2 (9, 10).

The genome of strain L2-7 consists of a single chromosome of 3,515,670 bp, with a G+C content of 38.6%. The genome of L2-7 is larger than the previously sequenced genome of *E. hallii* type strain DSM 3353, which has a size of 3.29 Mb (NCBI accession number PRJNA18177). The predicted number of coding sequences (CDSs) is 3,093. Furthermore, 24 rRNA genes and 72 tRNAs were identified, including 8 copies of the 16S and 23S rRNA genes. Investigation of the annotated genome revealed a complete pathway for the conversion of glucose to butyrate along with the gene encoding lactate dehydrogenase involved in converting lactate to pyruvate. We also detected genes for 1,2-propanediol conversion into propionate, in line with its metabolic properties (11). Moreover, the genome of strain L2-7 includes genes encoding a bile acid, sodium symporter (EHLA_2286) and choloylglycine hydrolase (EHLA_1602), suggesting its ability to tolerate and break down bile salts. In addition, we also detected genes involved in both the *de novo* and salvage pathways for biosynthesis of vitamin B_12_, which is an important cofactor for a variety of enzymes encoded by several intestinal bacteria and was recently shown to be functional in a trophic chain with *Akkermansia muciniphila* (12).

The genome of strain L2-7 adds to the increasing number of genomes for human intestinal bacteria and will contribute to an understanding of the physiology and potential health contribution of strain L2-7 in the human intestinal tract.

### Accession number(s).

The complete genome sequence of *Eubacterium hallii* strain L2-7 was deposited at GenBank/EMBL-EBI under the accession number LT907978 (assembly version EH1).
